# Callous–unemotional traits as a cross-disorders construct

**DOI:** 10.1007/s00127-012-0513-x

**Published:** 2012-05-09

**Authors:** Pierre C. M. Herpers, Nanda N. J. Rommelse, Daniëlle M. A. Bons, Jan K. Buitelaar, Floor E. Scheepers

**Affiliations:** 1 Karakter Child and Adolescent Psychiatry University Centre, Radboud University Nijmegen Medical Centre, Reinier Postlaan 12, 6525 GC Nijmegen, The Netherlands; 2 Department of Child and Adolescent Psychiatry, University Medical Centre Utrecht, P.O. Box 85500, 3508 GA Utrecht, The Netherlands

**Keywords:** Callous–unemotional traits, Juvenile psychopathy, Conduct disorder, Validity

## Abstract

**Purpose:**

Callous–unemotional (CU) traits are currently viewed as the defining signs and symptoms of juvenile psychopathy. It is unclear, however, whether CU traits have validity only in the context of conduct disorder (CD) as proposed by Frick and Moffitt (A proposal to the DSM-V childhood disorders and the ADHD and disruptive behavior disorders work groups to include a specifier to the diagnosis of conduct disorder based on the presence of callous–unemotional traits, American Psychiatric Association, Washington, DC, 2010), or also outside CD, either in combination with other forms of psychopathology or as a stand-alone construct.

**Methods:**

The current review systematically studied the existent literature on CU traits in juveniles to examine their validity inside and outside CD according to the framework regarding the validity of a psychiatric diagnosis provided by Robins and Guze (Am J Psychiatry 126:983–987, 1970).

**Results:**

Inside youth with conduct problems, and CD specifically, it seems that CU traits meet the Robins and Guze criteria. As many of the reviewed studies included youth with ODD and ADHD as well, there are indications the same might be true for ODD and ADHD, although probably to a lesser extent. In other disorders, CU traits may be present as well, but their role is not firmly established. As stand-alone construct, data are lacking or are scarce on all of the above-mentioned criteria.

**Conclusions:**

CU traits are a useful specifier in CD, and possibly also in disruptive behaviour disorders (DBDs) more generally. High CU traits outside DBDs exist but it is as yet unknown if there is a clinical need for defining CU traits as a stand-alone construct.

## Introduction

There is an increasing interest in the moderating role psychopathic traits may play regarding long-term outcome and treatment effectiveness of disruptive behaviour [1–3]. Specifically, there is a widely held belief that psychopathy has a poor outcome and is untreatable, although it seems more appropriate to state that a subgroup of patients with disruptive behaviour who portray psychopathic traits may require different treatment than patients with disruptive behaviour without these traits [2].

Although the history of the concept of psychopathy goes back to the nineteenth century [4, 5], today’s view on psychopathy is substantially based on Cleckley’s work, first published in 1941 [6], in which psychopathy is seen as a personality disorder. In recent literature, different aspects of psychopathy have been emphasized, such as: (1) disinhibition, poor impulsive regulation and the inclination to immediate gratification; (2) boldness, bravery, and thrill and adventure seeking; and (3) meanness, callousness and coldheartedness [7, 8]. There is now consensus that the presence of impulsive externalizing behaviour is not sufficient for a diagnosis of psychopathy but that boldness and/or meanness are the more typical characteristics. Particularly meanness is viewed by many experts as the core component of psychopathy. Frequently described symptoms of this core component are: lacking guilt and empathy, being very egocentric, showing callous use of others for ones own gain, and lacking normal emotionality, especially in showing a lack of anxiety. These symptoms have been known as callous–unemotional (CU) traits [1].

Regarding CU traits specifically, reviews have been published paying attention to the aetiology [9] and diagnostic value [10–12]. Some reviews have specifically focused on the conceptualization of CU traits in youth in relation to conduct problems [13–16], which is of importance because the aetiology and symptom presentation of a disorder in youth and adults may be different and need specific attention [5, 17, 18] and CU traits in antisocial youth seem to designate a distinct group that might develop into adult psychopathy. Furthermore, early detection might result into early intervention strategies preventing the development of adult psychopathy and antisocial behaviour. However, common to these reviews is that their focus is on the role of CU traits as a subtype of conduct disorder (CD) as proposed by the ADHD and Disruptive Behaviour Disorders Work Group for DSM-V [19] (see Table 1). Furthermore, in adults, subtypes of psychopathy can be distinguished. One of these subtypes is characterized by relatively high scores on deficient affective experience (comparable to CU traits) and low on antisocial behaviour [20]. Therefore, several questions remain: do CU traits represent a discrete or dimensional entity; are CU traits indeed related to CD only; does the psychopathological syndrome of CU traits show sufficient validity when assessed clinically (cf. [21])?

**Table 1:** Proposed specifier for callous–unemotional traits in the DSM-V

1.	Meets full criteria for conduct disorder
2.	Shows two or more of the following characteristics persistently over at least 12 months and in more than one relationship or setting.The clinician should consider multiple sources of information to determine the presence of these traits, such as whether the person self-reports them as being characteristic of him or herself and if they are reported by others (e.g. parents, other family members, teachers, peers) who have known the person for significant periods of time
	Lack of remorse or guilt: does not feel bad or guilty when he/she does something wrong (except if expressing remorse when caught and/or facing punishment)
	Callous-lack of empathy: disregards and is unconcerned about the feelings of others
	Unconcerned about performance: does not show concern about poor/problematic performance at school, work, or in other important activities.
	Shallow or deficient affect: does not express feelings or show emotions to others, except in ways that seem shallow or superficial (e.g. emotions are not consistent with actions; can turn emotions “on” or “off” quickly) or when they are used for gain (e.g. to manipulate or intimidate others)

http://www.dsm5.org/ProposedRevisions/Pages/proposedrevision.aspx?rid=424

By following the set of specific criteria for validation of psychiatric constructs, as proposed by Robins [22] and modified by Faraone [23], the current review aimed to contribute to the existing literature by taking a broader perspective on the nosological status of CU traits by focusing on their validity as a potential classifier for CD, other disorders than just CD and as a stand-alone construct (i.e. a separate DSM-diagnosis). These criteria are: (a) the construct has a consistent pattern of signs and symptoms, (b) the construct is dissociable from other related diagnoses, (c) the construct has a characteristic course and outcome, (d) the construct shows evidence of heritability from family and genetic studies, (e) data from laboratory studies demonstrate neurobiological and neuropsychological correlates of the construct, and (f) the construct shows a characteristic response to treatment. Since the Robins and Guze criteria were published more than 40 years ago, they may seem dated. Yet, they have been labelled as golden standard for establishing diagnostic validity, thus providing opportunity for psychiatric diagnoses to be defined as ‘real entities’ [24]. Recent papers have used these criteria for disruptive behaviour disorders [23, 25]. Yet, a few critical remarks can be made. The Robins and Guze criteria partially overlap with the set of criteria for construct validity, as formulated by Cronbach and Meehl [26], which has been of great importance as well [27]. Cronbach and Meehl [26] place an important emphasis on the nomological network, meaning that a construct needs to ‘function’ according to laws in which the construct occurs, while this criterion is not needed in the Robins and Guze criteria. However, the purpose of our paper was not to either investigate or extend the nomological network regarding CU traits, but to investigate the diagnostic validity of CU traits. Therefore, the Robins and Guze criteria seemed to fit better for our purposes. Another critical remark can be made regarding the fact that the Robins and Guze criteria do not seem to take comorbidity into account, yet comorbidity is the rule rather than the exception in mental disorders [28]. Therefore, it is relevant to clarify the distinctiveness of the relationship between CU traits and ‘established’ mental disorders. To our knowledge, this review is the first to apply these criteria to the construct of CU traits in youth.

A PubMed search was performed, focusing on research articles published between 1980 and December 2011, addressing CU traits as well as juvenile psychopathy, and review articles that appeared to be key articles (search terms: juvenile psychopathy and callous unemotional traits). Within the articles that were believed to be relevant, we have searched for additional literature. Studies had to use assessment instruments that quantified psychopathic and/or CU traits and had to have included comparison groups. CU traits were operationalized as those subdimensions of psychopathy which include symptoms such as callousness, shallowness and lack of empathy. This led to an initial 981 publications of which 206 (including 6 reviews) were eligible for this review. Findings are reported primarily in a qualitative manner.

## Criterion 1: Do CU traits have a consistent pattern of signs and symptoms?

The first criterion that must be met in order to consider CU traits as a construct inside or outside CD is that a consistent pattern of signs and symptoms must demarcate it from other disorders and from psychiatric wellness [22, 23]. However, there is no universal agreement with respect to how to best measure CU traits. CU traits seem to be a diagnostic construct that is still in a developmental stage.

The construct of CU traits has been developed on the basis of the concept of psychopathy. In the past 20 years, there has been an increasing interest in the concept of juvenile psychopathy and to our knowledge, at least 17 instruments have been developed which aim directly at assessing either psychopathic or CU traits, and which have been used in juvenile populations (see Table 2). The Psychopathy Checklist: Youth Version (PCL:YV; [29]) seems to be the first assessment tool that specifically focused on psychopathy in youth. Others followed and the versions for self-report were developed, leading to instruments such as the Antisocial Process Screening Device (APSD; [30]), and the Youth Psychopathic Traits Inventory (YPI; [31]). For reviews, we refer to [16, 32].

**Table 2:** Assessment instruments for measuring psychopathic traits in youth

Full name	Abbreviation	Reference	Rater
Psychopathy Checklist: revised	PCL-R	[228]	Clinician rated
Self-Report Psychopathy Scale: II	SRP-II	[229]	Self-report
Psychopathy Checklist: Screening Version	PCL:SV	[230]	Clinician rated
Survey of Attitudes and Life Experiences	SALE	[231]	Self-report
Childhood Psychopathy Scale	CPS	[232]	Clinician rated
Psychopathy Content Scale	PCS	[105]	Self-report
Psychopathy Screening Device	PSD	[233]	Self-report
Antisocial Process Screening Device	APSD	[30]	Self-report Teacher-report Parent-report
Youth Psychopathy traits Inventory	YPI	[31]	Self-report
Psychopathy Checklist: Youth Version	PCL:YV	[29]	Clinician rated
Inventory of Callous–Unemotional traits	ICU	[53]	Self-report Teacher-report Parent-report
Multidimensional Personality Questionnaire	MPQ	[141]	Self-report
Minnesota Temperament Inventory	MTI	[136]	Self-report
Social and Emotional Detachment Questionnaire	SEDQ	[74]	Parent-report
NEO Psychopathy Resemblance Index	NEO PRI	[234]	Self-report
Five Factor Model Psychopathy count	FFM PP count	[54]	Self-report Parent-report
Psychopathic Personality Inventory Short-Form	PPI-SF	[235]	Self-report

Trying to fractionate the concept of juvenile psychopathy, factor analyses have been applied on various instruments, mostly the PCL:YV, on the basis of scores obtained in community samples as well as in juvenile offenders. Although two-factor models [33–39], a four-factor model (e.g. [40–43]) and a five-factor model [44] have been proposed as underlying psychopathy with confirmatory factor analyses, a three-factor model seems to fit best (e.g. [44–51]). The three-factor model consists of factors which can be labelled as: (a) sensation seeking behaviour, (b) arrogant/deceptive interpersonal style, and (c) callous–unemotional traits. Discussion remains whether a fourth factor, labelled antisocial-aggressive behaviour, should be added [41, 42, 46].

The findings from these factor analytic studies are reasonably consistent in finding a distinct factor including lack of empathy, shallow affect and superficial interpersonal relationships, even though the factors are not always labelled similarly and their content may vary somewhat between different studies. This factor, with time increasingly called callous–unemotional traits, is consistently present in all models. This factor can be assessed reliably as from an age of 4 years [44]. Recently, a promising attempt was made to diagnose CU traits in preschoolers [52].

In youth, CU traits were increasingly seen as having incremental validity regarding diagnosing youth with conduct problems [19], which led to the development of the Inventory of Callous–Unemotional traits (ICU) [53], for assessment of CU traits specifically. Validation studies are promising, showing good internal consistency (Cronbach’s *α* = 0.69–0.83; [54–58] and concurrent validity (*r* ^2^ = 0.45–0.68 between ICU and APSD, and CPS) [57, 58]. However, other expressions of validity (e.g. temporal stability, interrater reliability) of the ICU specifically have to be established yet.

The current proposal to include a specifier for CU traits to conduct disorder in the DSM-5 formulates four criteria, of which two have to be met to assess CU traits (Table 1). For the development of this specifier, we refer to [19]. Internal consistency was shown to be moderate (Cronbach’s *α* = 0.56), yet many questions remain as it is unknown how well clinical validity is [19]. Thus, it seems that though CU traits as a construct show good ‘face validity’, the consistency of signs and symptoms within and specifically outside CD needs further evaluation.

## Criterion 2: Are CU traits dissociable from other related diagnoses?

A second criterion that must be met in order for CU traits to be considered as a valid nosological construct is its relative distinctiveness from other (related) DSM diagnoses. How often do high CU traits co-occur with CD? Are CU traits significantly more often linked to the presence of CD than to other disorders as ODD, ADHD, and ASD? Can high CU traits exist in the absence of other diagnostic entities, as CD, ODD, ADHD, ASD, personality disorder, mental retardation, and substance abuse (see Table 3)?

**Table 3:** Relationship between CU traits and specific diagnoses

Diagnosis	Support	No support
Conduct disorder	Core references: [35, 44, 59] Reviews: [15, 63]	
Oppositional-defiant disorder	[75]	[59, 74, 76]
Personality disorder	[236]	[55, 87–91]
Attention-deficit/hyperactivity disorder	[79, 80]	[81–85, 119]
Mental retardation		[81, 96–99]
Substance abuse		[101–105]
Autism spectrum disorder		[93, 94]
Anxiety disorder		
Self-reported anxiety ↓	[106, 107, 112, 113]	[33, 114]
Parent-rated anxiety ↓	[108–110]	
Teacher-rated anxiety ↓	[110]	
Clinician-rated anxiety ↓		[81]
Mood disorder		[116]

↓, decreased in the presence of high CU traits

### Disruptive behaviour disorders

Several epidemiologic studies investigated the prevalence of CU traits (see Table 4). CD was found to be present in 2 % of community children [59]. 32–46.1 % of community youth with CD was found to score high on CU traits [59, 60]. In the no CD group, 2.9–7 % scored high on CU traits [59, 60]. Comparable overall conclusions can be drawn from other epidemiological studies [19, 39, 61, 62] as well as a factor analytic study [35]. These studies imply that CU traits show overlap with CD, but are not interchangeable. Because of the upcoming CU traits specifier in the DSM-5, it is important to notice that numerous studies reported on a more pervasive and severe pattern of antisocial behaviour in CD patients with CU traits compared to those without CU traits. For reviews we refer to, e.g. [13, 15, 63], with recent publications confirming these observations [64–73].

**Table 4:** Prevalence of CU traits

Study	Sample	Male (%)	CD-only (%)	CD + CU (%)	CU-only (%)	CP + CU (%)	CP-only (%)
[62]	Community	51	n.i.	n.i.	7.2	5.6	7.9
[19]	Community	51–76	n.i.	n.i.	7–11	n.i.	
	Incarderated	51–76	n.i.	12–33	n.i.	n.i.	n.i.
[60]	Community	57	32 % of those with CD met criteria for specifier for CU traits 7 % of those without CD met criteria for specifier for CU traits				
	Clinic-referred	60	50 % of those with CD met criteria for specifier for CU traits 32 % of those without CD met criteria for specifier for CU traits				
[61]	Community	0	40.5 % reported at least once above 1 SD on CU traits over a 4-year period 65.5 % of the group meeting criteria for CD had high CU scores 44.3 % of the group with high CU traits met criteria for CD				
[59]	Community	–	1.1	0.9	2.9	n.i.	n.i.
[39]	Community	49	n.i.	n.i.	3.8	n.i.	
	Incarcerated	42	n.i.	n.i.	8.9	n.i.	

*CD* conduct disorder, *CP* conduct problems, *CU* callous–unemotional traits, *SD* standard deviation, *n.i.* no information given

Less is known about the possibility to use CU traits as a classifier in ODD. On the one hand, correlations between CU traits and ODD, and CD were found to be similar [74]; and CU traits have been described in youth with ODD only [75], suggesting CU traits may indeed be a useful specifier for ODD. On the other hand, CU traits are much more prevalent in CD than ODD [59, 76], making the clinical utility of a specifier in ODD less obvious. Further investigation regarding the relationship between CU traits and ODD specifically seems to be needed.

CD and ODD are both frequently comorbid with ADHD, making it relevant to examine the relationship between CU traits and ADHD. It has been argued [77] and demonstrated [78–80] that a subgroup of children with conduct problems and hyperactivity, impulsivity and attention problems (HIA-CP) resemble adult psychopathy. In contrast, several studies could not ascertain the relationship between CU traits and ADHD, when controlling for the presence of conduct problems [81–85].

Taken together, the presence of CU traits together with CD seems to lead to a specific syndrome with more severe antisocial behaviour, leading the DSM-5 workgroup to conclude CU traits are a useful specifier for CD. However, there are serious indications that CU traits are also present in youth with either ODD or ADHD without CD, albeit less prevalent, making the clinical utility of a specifier in ODD or ADHD less obvious. Regarding the validity of CU traits as a stand-alone construct, findings are scarce. High CU traits without disruptive behaviour do appear to exist based on several epidemiologic studies.

### Personality disorder

It has been argued that adult psychopathy is not only related to personality, but also that it is personality [86]. Much research in adult psychopathy has used personality questionnaires for delineating signs and symptoms of psychopathy. Findings show a strong negative relationship between psychopathy and agreeableness (expressing concern about interpersonal relationships and strategies) and conscientiousness (expressing ability to plan, organize, and complete behavioural tasks) [86]. As such, the question is whether CU traits in adolescents and adults are interchangeable with the personality disorders as described in DSM-IV-TR.

Several studies have investigated the relationship between CU traits and personality traits. These found an inverse association between CU traits with both agreeableness and conscientiousness [55, 87–89]. Furthermore, CU traits were found to be negatively associated with stress reaction (expressing reaction to distress, anxiety) and positively associated to aggression (expressing irritability, aggression) [90], and that CU traits in youth were not specifically related to narcissism [91]. Thus, these studies suggest CU traits to be related to certain personality dimensions/traits that can be apparent before the age of 18 years. However, none of these studies supports CU traits to be seen as equal to personality disorders.

Since personality disorders have their onset mostly in adolescence, and persist into adulthood [92], a next question is whether CU traits are predictors of adult personality disorders. However, we could not find any studies addressing this issue.

### Autism spectrum disorder and mental retardation

Because autism spectrum disorder (ASD) and mental retardation are both related to lower levels of empathy and self-reflection, it is possible that both are related to higher levels of CU traits. In youth with ASD, it was found that the correlation between severity of CU traits and ASD traits was extremely low, and callous antisocial behaviour did not appear to result from those cognitive deficits that are core to autistic disorders, such as ‘mindblindness’ and executive dysfunction [93]. Furthermore, boys with conduct problems and high CU traits were found to have dysfunctional affective empathy, but not cognitive empathy. The contrary was found in boys with ASD [94, 95]. Less is known about the relationship between intelligence (IQ) and CU traits. Some studies report no relationship [79, 96, 97]. Others report that youth with conduct problems and CU traits have a higher IQ [98] or in contrast a lower IQ [99] compared to youth with conduct problems without CU traits. Recently, CU traits were related to poor reading comprehension when controlling for IQ [79, 100]. These results suggest that autism and mental retardation are probably not related to CU traits, although both might hypothetically influence the phenotypic expression of CU traits.

### Substance abuse

Although the presence of psychopathic traits in substance-abusing adolescents is related to a higher level of alcohol- and drugs-related problems, there are no indications that CU traits in youth are the result of alcohol or drug abuse [101–105].

### Anxiety and mood disorders

As CU traits are associated with shallow affect and low fearfulness, anxiety was investigated in ten studies, of which seven controlled for conduct problems [81, 106–111]. CU traits mostly show a significant inverse relationship with subjective ratings of anxiety, either self-, parent-, teacher- or clinician reported [106–113], although this might account only for those children that perceived low levels of parental warmth/involvement [109]. Nevertheless, these correlations are not always found [33, 81, 114]. Mood disorders have been investigated scarcely. In a long-term follow-up study of about 10 years, mood problems in childhood were found to be predictive for CU traits in adulthood [115]. A recent study [116] investigated the relationship between CU traits and suicidality, and found no relationship in boys but an inverse relationship in girls, implying a protective role for CU traits. However, this latter study did not control for either conduct problems or CD. Therefore, there is insufficient information to draw conclusions regarding the relationship between CU traits and mood disorders.

## Criterion 3: Do CU traits have a characteristic course and outcome?

A third criterion for CU traits to meet the standards for a valid disorder is that they have a characteristic, and therefore predictable, course and outcome. This means that assessment of the diagnosis should lead to a clear prognosis. Especially important is the question: when CU traits are a subtype of CD, how strong do CU+ and CU− forms of CD differentiate from each other in external characteristics such as course and prognosis (see Table 5)?

**Table 5:** Characteristic course and outcome when CU traits are present in youth with conduct problems

Follow-up studies	Result	Support	No support
Short-term (0–4 years)	Social non-conformity ↑ Days detained ↑ Antisocial behaviour ↑ Symptoms of psychopathology ↑	[44, 112, 120, 124]	
	Substance use ↑	[122]	
	Proactive aggression ↑	[119, [118]; non-significant	
	General and violent recidivism ↑	[123] PCL:YV	[117, 123] APSD
	Delinquency ↑	[121]	
	Seriousness charges ↑	[110]	
	Impairment ↑	[61]	
	Stability of CU traits	[115, 130, 134, 137]	[140]
Long-term (4–12 years)	Severeness and chronicity of antisocial behaviour and delinquency ↑	[127–129]	
	Affiliation with deviant peers ↑	[125, 126]	
	CU traits show long-term stability	[52, 132, 135, 136, 139]	

[135], community twin sample; [52, 133], community sample; ↑, increased in the presence of high CU traits; ↓, decreased in the presence of high CU traits

Both short-term studies (up to 4 years) [44, 61, 110, 112, 117–124] and long-term studies (4–15 years) [115, 125–129] found CU traits to be predictive of more problematic behaviour. Studies investigating the long-term stability of CU traits showed this to be high over longer periods of time (i.e. 1 up to 53 years) [52, 115, 130–139]. Only a short-term study found stability to be low [140]. However, findings are comparable when no correction for conduct problems was made [110, 115, 128] and when a correction was made [119–121, 123, 126, 127], indicating the presence of CU traits quite robustly predicting a poorer outcome over and above the presence of conduct problems. The reviewed studies provide no information regarding CU traits as a stand-alone construct.

## Criterion 4: Do CU traits show evidence of heritability from family and genetic studies?

The next criterion to be met is whether it is possible to find evidence for a heritable nature of CU traits, supporting the hypothesis that CU traits are a valid entity, which can be delineated from environmentally caused psychopathology.

### Genetic influences

Genetic factors contribute importantly to the expression of CU traits [131, 135, 141–146], although environmental factors play a small-to-moderate role as well [131, 135, 142, 143, 145, 147] (see Table 6). Overlap with conduct problems is large, though not complete [145], indicating some room for unique genetic and environmental risk factors. High stability of CU traits seems to be related to high genetic influence [131, 135, 148], while antisocial behaviour seems to be more strongly related to unique environmental influences [142, 146, 147].

**Table 6:** Genetic influences on CU traits

Reference	*N*	Age (years)	Male (%)	Measure	Genetic influences (variance; %)	Shared environmental influences (variance; %)	Non-shared environmental influences (variance; %)	Genetic stability (variance; %)
[135, 141]	1,252 twins	Same group at 17 and at 24 years	46	MPQ	17 yr: 48 24 yr: 42		17 yr: 52 24 yr: 58	58
[148]	9,462 twins	Same group at 7, 9 and 12 years	47.3	3 items APSD + 4 items SDQ	78^a^ (boys) 0^a^ (girls)	1^a^ (boys) 75^a^ (girls)	2^a^ (boys) 25^a^ (girls)	
[131]	1,467 twins	Same group at 16 and at 19	40	YPI				82
[142]	2,198 twins	16–17	47.6	YPI	43	0	57	
[143]	398	16–18	100	MTI	42		58	
[144]	832 twins 1,056 probands	6–8	n.i.	3 items APSD + 4 items SDQ	67	6	27	
[145]	3,196 twins	7	48.0	Idem	67 (boys) 48 (girls)	4 (boys) 20 (girls)	29 (boys) 32 (girls)	
[146]	464 twins 314 probands	9	n.i.	Idem + 2 items ICU	75	0		
[147]	4.508 twins	Same group at 7 and at 12	n.i.	3 items APSD + 4 items SDQ	7 yr/P: 63 12 yr/P: 81 7 yr/T: 71 12 yr/T: 56		7 yr/P: 27 12 yr/P: 23 7 yr/T: 31 12 yr/T: 40	

*MPQ* Minnesota Personality Questionnaire [141], *YPI* Youth Psychopathic Traits Inventory [31], *MTI* Minnesota Temperament Inventory [136], *APSD* Antisocial Process Screening Device [30], *SDQ* Strengths and Difficulties Questionnaire [237], *Yr* year, *P* parent rating, *T* teacher rating

^a^Standardized estimates when stability over time was high

In recent studies, focus has not only been directed on phenotypes in twin studies, but also on molecular genetic underpinnings of CU traits as well. Up till now, the few genetic studies at a molecular level implicate multigenetic influences and a gene by environment interaction [149–151]. Possible positive associations have been described for gene variants that affect monoamino oxidase A (MAO-A), catechol-*O*-methyltransferase (COMT) [150], and serotonin transporter (5HTT) [150, 151]. None of these molecular genetic studies investigated CU traits outside either conduct problems or CD, making it uncertain whether the genetic findings are specific for CU or more broadly associated with disruptive behaviour disorders (DBDs).

### Environmental influences

Social and biological environmental factors play a small-to-moderate role as well (see Table 7). *Parenting style* is found to be negatively related to later developing CU traits [62, 133, 152], as well as positive parenting was found to be related to decreased CU traits [130, 153]. Furthermore, maternal CU traits, resulting in parental hostility and parenting dysfunction, play an important role in the intergenerational continuity of maternal CU traits [152]. In contrast, others suggested that an ineffective parenting style is unrelated to the presence of callousness [154], or only for children with low levels of CU traits [108, 155–157]. Also it is suggested that high CU traits may lead to reduced monitoring behaviours of parents [121], increased parenting stress [158], decreased eye contact towards mothers [159], and to decreased parental involvement towards boys [130]. Furthermore, social economic status is generally found to be lower in children scoring high on CU traits [62, 115, 133, 160, 161]. Interestingly, prenatal risk factors such as maternal problems [62] and tobacco use [115] were found to be a significant predictor for CU traits as well. Finally, several studies indicate that traumatisation [109, 162–164], as well as disorganized attachment [165], and early institutional deprivation [166] can be related to CU traits as well. This leads to the overall impression that the role of environmental influences is present albeit somewhat inconsistent, which might be due to the correlational nature of these studies. Furthermore, only a few studies controlled statistically for conduct problems [114, 115, 152, 153], and none for CD, making it uncertain whether the findings are completely accounted for by the presence of CU traits instead of conduct problems more generally. Nevertheless, social and biological environmental influences might play a role in CU traits co-occurring with CD. Unknown is the role of the environment when CU traits exist independently of other psychiatric disorders.

**Table 7:** Correlational environmental studies

Type of environmental influence	Support	No support
Parenting style (−)	[62, 130, 133, 152, 153, 165, 166]	[108, 121, 154–159]
SES (−)	[62, 115, 133, 160, 161]	
Physical traumatization (+)	[109, 162–164]	
Prenatal risk (+)	[62] Prenatal maternal problems [115] Exposure to cigarette smoke	

−, inverse correlation with CU traits; +, positive correlation with CU traits

## Criterion 5: Do CU traits have specific and differentiating neurobiological and neuropsychological correlates?

The fifth criterion requires the presence of specific neurobiological correlates of a disorder [23]. Psychological tests, when shown to be reliable and reproducible, may also be considered laboratory studies in this context [22]. The literature on CU traits is too extensive to provide a detailed review. Nevertheless, we will provide a comprehensive summary (see Table 8).

**Table 8:** Neurobiological and neuropsychological studies on CU traits

	Focus	Support	No support
Neurobiological markers	fMRI: Amgdala ↓ vmPFC ↓	[167–169]	[170] ?
	sMRI: Amygdala =	[171]	
	Cortisol ↓	[76, 115, 173]	[172] ?
	Testosteron =	[173]	
	Skin Conductance ↓	[57, 175, 177, 178]	
	Heart rate ↓	[174, 176]	[52] Preschoolers
Prosocial reasoning	Prosocial reasoning ↓	[179–182]	[161, 190, 191] ?
	Cognitive, but not affective empathy improves during growth	[94, 95, 189]	
	Egoistic functioning and acceptance of social deviant behaviour	[183–185]	
	Positive labelling of aggression and acceptance of social deviant behaviour	[186–188]	
Reward sensitivity	Reward sensitivity ↑ Impulse inhibition ↓ Punishment avoidance ↓	[81, 161, 184, 192, 193]	
Emotional reactivity	Emotional reactivity ↓	[52, 56, 164, 194]	
	Self-reported arousal ↓	[83]	[195]
Emotion recognition	Recognition of fear ↓	[196–204]	
	Recognition of sadness ↓	[95, 196, 202, 203, 205, 206]	

↑, increased in the presence of high CU traits; ↓, decreased in the presence of high CU traits, =, no differences between high CU traits and control; ?, results inconsistent

### Neurobiological markers

Studies on neurobiological markers are important to investigate whether it is possible to find neurobiological underpinnings for CU traits. Functional magnetic resonance imaging (fMRI) studies suggest impaired functioning of the amygdala [167, 168] and of the ventromedial prefrontal cortex (vmPFC) [169], as well as weaker functional connectivity between these two brain areas [168] in youth with conduct problems and CU traits, compared to healthy controls. The only fMRI study controlling for CD could not detect correlations with CU traits [170]. However, this study used only pictures of angry, sad and neutral, but not fearful faces. As discussed below, decreased recognition of fearful faces is most strongly related to CU traits, compared to other types of faces. Furthermore, using structural MRI in a comparison between normal control children with children scoring high on conduct problems and CU traits increased grey matter in several brain areas, specifically in the PFC, has been found, but not in the amygdala [171].

As CU traits are associated with decreased anxiety, and cortisol levels are associated with anxiety levels, it is expected that increased CU traits correlate with decreased cortisol levels. One study could not detect this relationship, however probably because the level of CU traits was still low in the CU traits group [172]. In contrast, three studies did find blunted [76] or decreased baseline [115, 173] cortisol levels to correlate with CU traits.

Electrophysiological studies are important because CU traits are thought to relate to lower physiological arousal. Indeed, these studies show lower physiologic responsiveness in youth with CU traits, compared to youth without these traits, specifically in reaction to distress, and provocation [57, 120, 124, 174–178], although in preschoolers with high CU traits, higher overall physiological arousal was found [52].

Three studies controlled for CD [115, 170, 174], one for ODD [52], and several others for conduct problems [57, 76, 172, 173, 176–178]. Therefore, CU traits appear specifically related to the above-mentioned neurobiological abnormalities. Nevertheless, since no studies have examined neurobiological abnormalities in individuals with CU traits without any psychiatric disorders, it is difficult to infer conclusions about the relationship between neurobiological markers and CU traits outside CD.

### Prosocial reasoning

Studies on prosocial reasoning frequently use stories about hypothetical problematic social situations in which one has to solve moral dilemmas. Three studies specifically controlled for CD [94, 95, 179]. Less prosocial reasoning was found in youth with CD-high CU than in youth with CD-low CU and normal controls [179], while cognitive functioning seems to be unimpaired [94, 95]. Except for two [180, 181], the following studies controlled for conduct problems. Less prosocial reasoning was found as well in youth with conduct problems and high CU traits than in those with low CU traits [180–182], as well as more egoistically functioning, more problems in affective perspective taking, accepting more social deviant behaviour [183–185], and specifically aggression [186–188]. Furthermore, the presence of conduct problems together with CU traits has been associated with deficits in affective empathy, specifically for boys at different ages [95, 189], and improvement of cognitive empathy through the pubertal years [189]. A few studies seemed to find contradictory results, such as less reactivity and hostility [161], decreased expectations regarding the use of aggression [190], and more proactive behaviour [191] in the presence of high CU traits. However, these findings [161, 190, 191] can be explained by an increased orientation towards achieving ones goals. Thus, the reviewed studies support the notion of decreased affective prosocial reasoning in relation to increased CU traits over and above the relation of affective prosocial reasoning to conduct problems. However, virtually no information on prosocial reasoning is available regarding CU traits outside CD.

### Reward sensitivity

Findings on emotion processing have focussed on inhibition deficits, anxiety and response-to-distress as can be shown by psychological tests. These studies suggest a response modulation deficit in which a greater reward sensitivity, accompanied with decreased impulse inhibition and punishment avoidance play an important role when CU traits are high in youth with conduct problems [81, 161, 184, 192, 193]. These studies controlled for conduct problems, although not for CD, suggesting abnormal reward sensitivity is related to the presence of CU traits over and above the presence of conduct problems. We found no studies regarding CU traits outside conduct problems.

### Emotional reactivity

Emotional reactivity refers to the extent in which participants react to psychological discomfort. Four studies in youth with conduct problems [52, 56, 164, 194], with one controlling for CD [194], and one for ODD [52], suggest that CU traits are associated with lowered emotional reactivity in laboratory tests. In contrast, CU traits were not found to be associated with self-reported arousal in a community sample [195], although in a later study, based on the same community sample, high CU traits correlated with decreased self-reported arousal ratings to unpleasant pictures [83]. Thus, CU traits seem to be related to decreased emotional responsiveness, most likely over and above the presence of conduct problems.

### Emotion recognition

In the presence of CU traits, the most consistent findings are impaired recognition of fearful faces in community youth [196–200], clinic referred youth [201], and youth with conduct problems [202, 203]. Directing attention to the eyes seems to improve facial emotion recognition [197, 198]. Furthermore, adolescents with conduct problems and high CU traits showed consistent impairments in eye contact to their parents, while higher levels of eye contact between fathers and their sons were associated with better fear recognition [204]. Findings on recognition of sadness are less consistent [95, 196, 202, 203, 205, 206]. All, but two studies [196, 200] controlled for conduct problems, and three for CD specifically [95, 203, 205], indicating that abnormal emotion recognition seems robustly associated with CU traits over and above the presence of abnormal emotion recognition in relation to conduct problems. Again, we found no studies regarding emotion recognition in CU traits outside conduct problems.

## Criterion 6: Do CU traits show a characteristic treatment response?

Response to treatment is the last criterion when viewing the validity of a diagnosis. Accurate diagnosis is important, because it determines the success of treatment. Vice versa, the need for a specific treatment for a specific disorder confirms its validity. To date, there seem to be only few studies that focused on improving treatment response in youth with CU traits (see Table 9).

**Table 9:** Treatment response in youth with conduct problems moderated by the presence of CU traits

Reference	*N*	Age (years)	% male	Diagnosis	Measure	Treatment type	Clinical improvement on behaviour	Length of treatment	Treatment compliance	Follow-up after treatment
[208]	248	12–14	100	Conduct problems	PCL:YV	Residential treatment for adjudicated youth with severe conduct problems	High CU = low CU			
[214]	69	11–17	60	Conduct problems	APSD mCPS	Juvenile diversion program			Program failure → high CU > low CU	Rearrest at 1 year → high CU > low CU
[209]	70	6–13	34.3	ODD or CD and ADHD (77.1 %) No Dx (22.9 %)	APSD	Summer treatment program	Social skills and problem solving → high CU < low CU		Negative behaviours in time-out → high CU < low CU	
[210]	56	4–8	n.i.	ODD (and conduct probems; and secondary ADHD)	APSD	Parent training	Disciplinary measures → high CU < low CU	Treatment sessions → high CU > low CU		Outcome at 6 months after treatment → high CU < low CU
[211]	56	4–8	n.i.	ODD (and conduct probems; and secondary ADHD)	APSD	Parent training				In stable-high CU group → most severe conduct problems at 6 months
[212]	177	6–11		ODD or CD (and ADHD)	APSD	Modular treatment at (1) outpatient clinic, (2) at home and school and (3) treatment as usual	High CU = low CU			High CU = low CU
[213]	38	6–14	73.7	ODD/CD	APSD ICU	6-month therapeutic program including cognitive behavior programs	High CU < low CU			
[215]	64	15–19	100	Substance abuse	PCL:YV	Substance abuse program for adjudicated adolescents	High CU < low CU		High CU < low CU	Rearrest rate → high CU > low CU (*p* < .10)
[216]	85	11–18	100	Conduct problems	PCL:YV APSD mCPS	Residential treatment for adjudicated youth	Physical incidents → High CU > low CU (APSD; mCPS) Back to lower level of treatment → high CU > low CU (mCPS)	Days of treatment to reach next level → high CU > low CU (mCPS) high CU = low CU (APSD)		
[217]	100	7–17	66	ODD or CD (68 %) Other Dx (32 %)	APSD	Psychiatric hospitalization		Length of treatment → high CU > low CU		
[207]	37	7–13	78.4	ADHD/ODD (43.2 %) ADHD/CD (56.8 %)	APSD	RCT: BT + Placebo vs. BT + MPH	BT + Placebo → high CU < low CU BT + MPH → high CU = low CU		Compliance → high CU < low CU (marginally)	

*RCT* randomized controlled trial, *BT* behaviour therapy, *MPH* methylphenidate, *ADHD* attention deficit hyperactivity disorder, *CD* conduct disorder, *Dx* diagnosis, *ODD* oppositional-defiant disorder, *n.i.* no information given, *PCL:YV* Psychopathy Checklist: Youth Version [29], *APSD* Antisocial Process Screening Device [30], *mCPS* modified Child Psychopathy Scale [232]

We found one study [207] applying a placebo-controlled treatment design. In this study, the response to behaviour modification with and without methylphenidate was examined. Boys with ADHD, conduct problems and high CU traits did not improve as much with behavioural therapy as those with low CU traits. However, when treated with methylphenidate, these differences largely disappeared, suggesting a beneficial effect of methylphenidate.

Other studies described treatment effects in open designs, of which seven explicitly controlled for conduct problems [207–213] or CD and ODD [207, 208, 211, 212]. Except two [208, 212], most open studies suggest a negative effect of CU traits, over and above conduct problems, on either treatment progress, outcome or follow-up [208–210, 213–217]. Data are lacking on treatment effect of CU traits in the presence of other disorders than DBDs.

## Summary and conclusion

This review examined the nosological status of CU traits by focusing on their validity in children and adolescents not only as a subtype of conduct disorder, but also as a potential classifier for other disorders or as a stand-alone construct. CU traits may moderate the treatment of disruptive behaviour disorders and the categorization of patients with these traits could be helpful in developing adequate therapeutic interventions. This topic was addressed by applying criteria for validation of psychiatric diagnoses, as formulated by Robins and Guze [22], and modified by Faraone [23].

Based on the reviewed studies, we conclude that the presence of CU traits can be assessed reliably as from school age, with preliminary data suggesting reliable assessment at preschool age as well. Although assessment measures are still in development, a consistent pattern of signs and symptoms is found demarcating it from other disorders. Furthermore, CU traits are associated with a distinct pattern of conduct problems in CD, while there are indications that the same might be true for ODD and ADHD. That is, the presence of CU traits is related to a more aggressive and more pervasive kind of conduct problems. Similarly, CU traits can be distinguished from other psychiatric diagnoses in juveniles, such as autism spectrum disorder, mental retardation, personality disorder, substance abuse, and mood and anxiety disorders. In addition, there is a characteristic course and outcome: the presence of CU traits in youth with disruptive behaviour is increasingly stable with the increase of age and associated with increased levels of conduct problems, delinquency, reoffense and/or substance use over longer periods of time from childhood up to adulthood. Moreover, as antisocial behaviour decreases with aging, CU traits persist through life. In twin studies, genetic influences are shown to account for 43–81 % of CU traits. Furthermore, social and biological environmental influences such as poor parenting and traumatisation were found to cause a detrimental effect. Neurobiological and neuropsychological correlations can be found, in which findings indicate decreased prosocial reasoning, decreased responsiveness to distress cues, and decreased recognition of fearful and perhaps sad faces in youth with CU traits. Furthermore, in youth with conduct problems and high CU traits biological differences can be detected as well, such as impaired amygdala functioning, impaired functioning of the vmPFC, impaired connectivity between these two brain areas, as well as decreased cortisol levels and physiological arousal. Finally, treatment requires specific attention in the presence of CU traits: conduct problems are more severe at the start of the treatment, response to behavioural treatment is worse, and a more intensive treatment is required before improvement can be observed. Thus, there is clear supportive evidence for CU traits as a valid subtype of CD. Hence, we believe that CU traits are a valid and viable sub diagnosis, which give the opportunity to make an important differentiation especially in different kinds of conduct problems and antisocial behaviour. Moreover, given that the majority of studies were conducted in youth with conduct problems (i.e. ODD and CD grouped together) and several studies indicate that CU traits and ODD and to a lesser extent ADHD seem to be correlated as well, we believe CU traits may be a useful specifier for DBDs in general. No compelling evidence exists for CU traits as useful specifier in other psychiatric axis I and II disorders. Furthermore, although many of the above studies controlled for the presence of conduct problems, it still is difficult to mark CU traits as a stand-alone construct. Therefore, the question remains whether CU traits outside conduct problems constitute a clinical problem or not.

## Future research

As this review covers a broad range of topics, related to the validity of CU traits, many issues for further research emerge. However, as others have pointed out the importance of further research to determine how the criteria for CU traits can be incorporated in the DSM in a valid and useful way [218, 219], in this paper we will specifically address points for future research concerning the validity of CU traits for DBDs in general and as a stand-alone construct.

This review shows that much research has been done in children and adolescents which supports the importance of distinguishing CU traits as an important symptom cluster in addition to conduct problems. We found many studies that combined youth with ODD and youth with CD into a single study group when investigating the moderating role of CU traits, mostly because ODD and CD are reasoned to reflect a single conduct domain (e.g. [136]). Nevertheless, there is still discussion whether these diagnoses represent the same underlying entity, and that ODD symptoms should not be seen as a milder, earlier presentation of CD [220]. Therefore, it is important to further investigate the relationship between CU traits and ODD specifically.

This leads us to another important issue. In the vast body of literature, we found only five studies, explicitly reporting on CU traits when scoring low on conduct problems [61, 75, 126, 127, 161]. As the findings from these studies are contradicting, and as the prevalence of CU traits in community samples seems to be relatively high, it seems to be important to direct further research on CU traits outside CD, and in the absence of a disruptive disorder diagnosis. Thus, the relevance of CU traits over and beyond either CD and ODD will become much clearer. Through gathering clearer epidemiologic data, we might improve our knowledge about the overall prevalence of CU traits, identify aetiologic factors, and help to estimate the need for services [221].

Further research on the conceptualization of CU traits seems needed as well. Three issues seem to need further attention specifically. First, increased consensus about diagnostic criteria is needed. In this review, we encountered many different conceptualizations of CU traits. However, the proposal of Frick and Moffitt [19] to include CU traits as a specifier to the diagnosis of CD in de the upcoming DSM-5 is especially meaningful for this issue. Second, as there are indications for specific differences between boys and girls regarding either psychopathic or CU traits (e.g. [103, 222–224], gender issues seem to need more attention as well. Third, as Hong Kong children were found to have higher scores on CU traits than United States children [45], cultural issues might play an important role as well. Hence, it is important to invest in specification of diagnostic criteria, which take gender and cultural differences into account.

Effective treatment in youth could dramatically reduce violent incidents and victim injury [225]. Although promising results have been shown with interventions that aim at improving prosocial behaviour using positive reinforcement, either by parent training, training of individual social abilities, or medication, still there is a need for further randomized controlled treatment trials in youth with conduct problems and CU traits regarding short-term as well as long-term treatment effects. Also research seems to be warranted on the question whether specific treatment is needed in the presence of CU traits outside conduct problems.

Then there is the topic that, although several theories about developmental pathways regarding psychopathy and CU traits have been proposed [2, 164, 226], the ultimate causes in terms of gene-environmental interplay as well as (deviancies in) brain development have to be unravelled. This leads to several questions, such as whether it is possible to extract candidate genes for further genetic research, while new types of genetic research, such as imaging genetic work, seem to be promising in clarifying the role of specific endophenotypes [9]. However, as parenting practices are among the most powerful predictors of later outcomes in children and constitute opportunities for interventions [227], a next question is whether specific parenting practices at young age might decrease the further development of CU traits either in- and outside CD. Finally, the MRI studies raise further questions about the underpinnings of CU traits. Although it is hypothesized that there is a distinct brain development in boys with callous–unemotional conduct problems [171], this needs further exploration.

This review has focused on the diagnostic validity of CU traits in- and outside CD. It becomes clear that CU traits have gained increasing attention in the past years, and our understanding on this topic increases steadily. However, much research is needed on the prevalence, aetiology, and need for diagnosis and treatment of CU traits outside CD, to improve our understanding of CU traits as a cross-disorders construct and possibly as a stand-alone construct as well.
